# Current evidence on the cell of origin of meningiomas disputes the arachnoid cap cell dogma

**DOI:** 10.1007/s00701-026-06908-1

**Published:** 2026-05-21

**Authors:** M. Necmettin Pamir, Ayça Erşen-Danyeli, Koray Özduman

**Affiliations:** 1 Departments of Neurosurgery, Acıbadem University, School of Medicine, Altunizade, Yurtcan Sokağı No : 1, 34662 Üsküdar, İstanbul, Turkey; 2 Pathology, Acıbadem University, School of Medicine, İstanbul, Turkey

**Keywords:** Meningioma, Arachnoid cap cell, Ontogeny

## Abstract

**Background:**

For over a century, the dominant hypothesis has maintained that meningiomas arise exclusively from arachnoid cap cells. However, emerging evidence from single-cell transcriptomics, spatial sequencing, developmental biology, and transgenic animal models suggests a significantly more complex and heterogeneous cellular origin.

**Methods:**

This mini-review revisits historical perspectives and critically evaluates the limitations of the traditional arachnoid-based paradigm. By synthesizing classical histological insights with contemporary molecular and proteomic data, the study explores alternative hypotheses, particularly those implicating fibroblast-like or regionally distinct meningeal progenitors.

**Results:**

The synthesis of multi-omic and developmental data indicates that a singular cellular source is insufficient to account for the clinical and biological diversity of meningiomas. Findings suggest that meningioma ontogeny is likely driven by a broader spectrum of progenitors whose characteristics vary according to anatomical location and embryological lineage.

**Conclusions:**

A redefinition of meningioma ontogeny is necessary. Future classification and therapeutic strategies should reflect the inherent diversity of their cellular sources and anatomical environments, moving beyond the classical arachnoid-cap-cell model.

## Introduction

The ontogeny of a tumor, in other words “the cell of origin”, provides the scaffold that is necessary for diagnosis, risk stratification, prognostication, therapeutic targeting and experimental modelling for a tumor. It provides information on the etiology as it points to the specific vulnerability allowing the initial oncogenic change to occur, and very importantly its timing. Therefore, it has been in the focus of meningioma research for over a century.

In almost all textbooks, “arachnoid cap cells” are cited as the “cell of origin” for meningiomas. Having originated from early reports of John Cleland and Martin Schmidt and subsequent support from Harvey Cushing, this hypothesis has dominated the literature, despite very limited scientific evidence [13]. Novel findings and experimental evidence is slowly challenging this dogma and changing our understanding. The current evidence points to a more diverse set of cells rather than one single cell of origin. The current literature also supports the notion that such cells or origin are likely early progenitors that exist early during development of the meninges, secondarily shaped by the tumor microenvironment.

## The evolution of meningioma nomenclature

The precise cellular and locational origins of meningiomas have consistently been questioned for almost three centuries. In 1774, Antoine Louis in France referred to dural-based tumors as “fungating tumors of the dura mater”, offering one of the earliest tumor descriptions linked to meninges [31]. This perspective was reinforced by Jean Cruveilhier in 1835, who differentiated between “bloody tumors- “ and “cancerous tumours-of the meninges” [12]. Cruveilhier’s description indicated to a histologically defined localization. Hermann Lebert published 21 of his cases and 80 others that he collected from the literature and published the first large study on this tumour type [27]. Lebert called the tumour “Tumeurs fibro-plastiques intra-craniennes” and also differentiated between “cancerous” and “non-cancerous” forms in 1851. Sir James Paget mentioned this tumour type in his book on cancers in 1954 and defined these as myeloid (marrow like) tumours and noted that they are less malignant than other cancers [35]. In 1863 Rudolf Virchow identified small calcifications within the tumour and called the tumour “psammoma” and its malignant form “dural sarcoma” [35]. In 1869 Camilo Golgi named these dural based tumours as “dural endotheliomas” based on the Wilhelm His’ idea that serous cavities and the meninges should be considered “endothelial” [35]. In the 1920 s, Pío del Río-Hortega introduced the term “exothelioma” based on the characteristic external covering function of arachnoid cells, arguing that earlier terms such as “endothelioma” were inappropriate and misleading [35]. Around the same period, Victor Cornil and Louis Ranvier described vascular variants as “angiolytic sarcomas,” suggesting a blood vessel–associated mesenchyme as the source. The current “meningioma” name was given by Harvey Cushing, who in his 1922 Cavendish lecture noted that this tissue based name was a simple and non-committed designation, which did not suffer from the limitations of an ontogenic or anatomic nomenclature [13]. However, as it will be more evident in the rest of this text, meningiomas exhibit considerable variation among them (anatomically, pathologically, genetically and clinically) and this is to such a degree that it would not be absurd to define them as a “group of tumours” (similar to the example of “gliomas”) rather than a discrete tumour entity.

## How the evidence on meningioma ontogeny developed

### Origin of the “arachnoid cap cell origin theory”

In the early years of the twentieth century, pathologists had conflicting ideas on the origin of meningiomas. Ribbert postulated that meningiomas originated from connective tissue, Mallory from fibroblasts, Oberling from glia, Roussy and Cornil from neuro-epithelial cells [35]. Among those different theories, the one that gained traction was on the origin from arachnoid granulations. Richard Bright from Edinburgh University had given one of the first reports of meningiomas and had pointed to the arachnoidal lining of the dura as the origin of the disease without giving a specific name to the disease in 1831 [35]. In 1864 when John Cleland, a Scottish anatomist and surgeon from University of Glasgow, reported his results on a frontal convexity and an olfactory grove meningioma, he defined them as “villous tumours of the arachnoid” and concluded that they should arise from Pacchionian corpuscles [10]. Shortly thereafter C. Robin also reported two meningeal tumours in 1869, which he attributed to arachnoid origin [35]. These two conclusions were not completely new, but Luschka in 1852, Meyer in 1859, Key and Retzius in 1876 had hinted to similarities between arachnoid granulations and meningiomas. A more detailed work by Martin B. Schmidt in 1902 concluded that the tumours should originate from endothelial cell clusters capping the arachnoid villi [43]. Aoyagi and Kyuno [2], expanded on this anatomical perspective by examining dura mater samples across age groups, identifying endothelial cell processes that supported an arachnoid-derived origin in 1912. A pivotal moment came in 1911 with Cushing’s 15th case, which suggested that these tumours originated from the parasagittal sinus wall and not the dura proper. Harvey Cushing presented the case of a lady with a paramedian convexity meningioma, who also had arachnoidal cell whorls located outside the tumour in the falx cerebri in his 1922 Cavendish lecture and supported the “arachnoidal cap cell origin theory” [13]. Cushing used this theory to explain the “Favored seats of origin of meningiomas, which were not random but rather reflected localizations where arachnoid villi are most abundantly developed. He considered the dura to be a reactive host rather than a site of tumour origin. Cushing’s view, although grounded in meticulous anatomical observation, relied on a largely static and partial understanding of meningeal biology. He noted, for example, the fibrous nature of some meningiomas and their capacity to infiltrate bone or dura yet interpreted these traits as reactive phenomena rather than indications of lineage diversity. His language, such as “psammomatous transformation” and “meningotheliomatous cell,” reflected an attempt to categorize based on morphology without developmental insight.

**Fig. 1:**
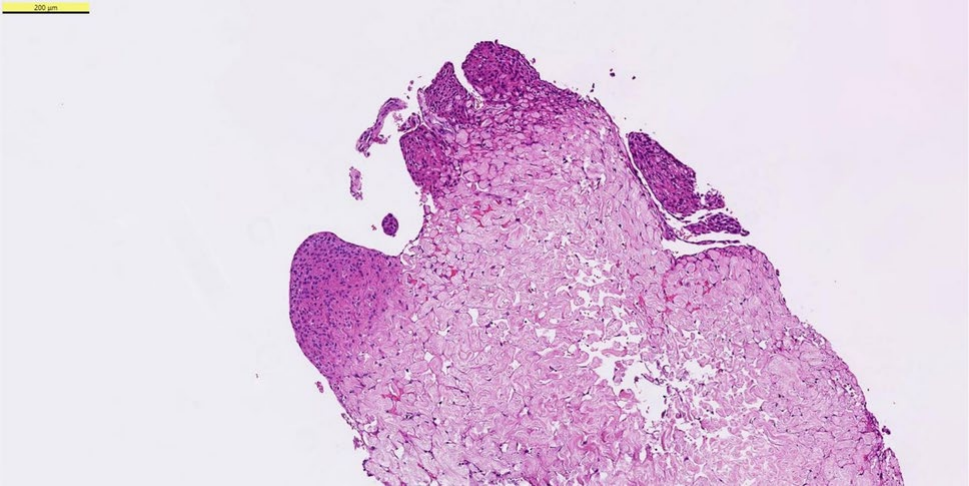
Arachnoid cap cells are specialized cells, that are located at the top of arachnoid willi

Despite the lack of concrete scientific evidence the “arachnoid cap cell origin theory” became the dogma and dominated the twentieth century. Cytological similarity between arachnoid villi and meningiomas, whorl and psammoma formation, the location around venous sinuses (, which correlated with the favored sites of origin,) shared epithelial membrane antigen (EMA) and vimentin expression supported this hypothesis. Nevertheless, it was well recognized from the beginning on that the theory was short of explaining the variability in histology, wide distribution of meningiomas and unexpected localizations such as the intraventricular site of meningiomas. The anatomical distribution of arachnoid villi—and by extension, cap cells—is regionally limited (Fig. 1). They are virtually absent from many intracranial compartments where meningiomas frequently arise, such as the anterior cranial fossa, the convexity dura, and critically, intraventricular locations. In these areas, arachnoid villi are either underdeveloped or entirely absent, raising fundamental questions about the plausibility of arachnoid cap cells as the universal cell of origin.

### How did our understanding on the “origin of cancer” evolve?

When naming and classifying meningiomas, Cushing avoided an “ontogenic” classification. He argued that such was not possible due to lack of clear, undisputed knowledge in the development of meninges [13]. His approach to other tumours was different. Bailey and Cushing were the first to focus on ontogeny to classify gliomas in 1926. This classification was based on “Julius Conheim and Hugo Ribbert’s theory of cancer” which was the widely accepted notion of that day [6]. Coheim and Ribbert’s hypothesis proposed that cancers developed from heterotopic nests of embryonal cells that are triggered by chronic inflammation or trauma to form tumours. In the first half of the twentieth century, the hypothesis of a “block in differentiation” for central nervous system tumours was challenged by authors such as Hans Joachim Scherer and Nils Ringertz, who favored the alternative hypothesis of “de-differentiation” [42]. As a block in embryological diferentiation could explain the formation of a tumour but could not explain increasing malignancy over time. Their theory of de-differentiation claimed that the terminally differentiated cells of the tissues would undergo genetic alterations to form tumours. Soon, molecular biology provided solid evidence on how cells underwent oncogenic transformation by accumulation of genetic alterations [44]. Such “oncogenic programs” were proven to transform normal cells into cancerous counterparts. Nevertheless, an explanation on the cell of origin was still lacking. Subsequent studies showed that the “cell of origin” also had enormous influence on the final cancer phenotype, independent of the cancer program [21]. The next great leap in our understanding came with the identification of “cancer stem cells” in various central nervous system tumours [46]. Such cells exhibited various traits of stem cells and could re-populate large tumours starting from a very small number of cells. Every human tissue physiologically has progenitor cells, which are responsible for housekeeping and regenerating that tissue. The current understanding is that accumulation of genetic alterations (the oncogenic programs) results in the transformation of resident progenitors. Mesenchymal stem-like cells as well as stem-like gene expression programs have been discovered in meningiomas, but their role and function still need further study [4, 28]. Finally, the tumour development process is influenced by the host organism (e.g. The host immune system) to result in a complex tumour microenvironment. In the case of meningiomas, the oncogenic programs have been largely uncovered but the cell of origin and the effects of the tumour microenvironments are still to be clarified.

### Meningioma is not a homogenous disease

Early studies by Cruveilhier, Lebert, and Virchow had clearly demonstrated the presence of benign and malignant tumour types [35]. Bailey and Bucy’s 1931 publication described 9 different types of meningiomas, which they claimed to arise from different intermediate cell types [3]. The current 2021 WHO classification of central nervous system tumours differentiates 15 meningioma subtypes. More recent studies also point to 6 distinct methylation subsets and 7 distinct transcriptomic subsets [41, 48]. It is very unlikely that these diverse subtypes have a single cell of origin.

**Fig. 2:**
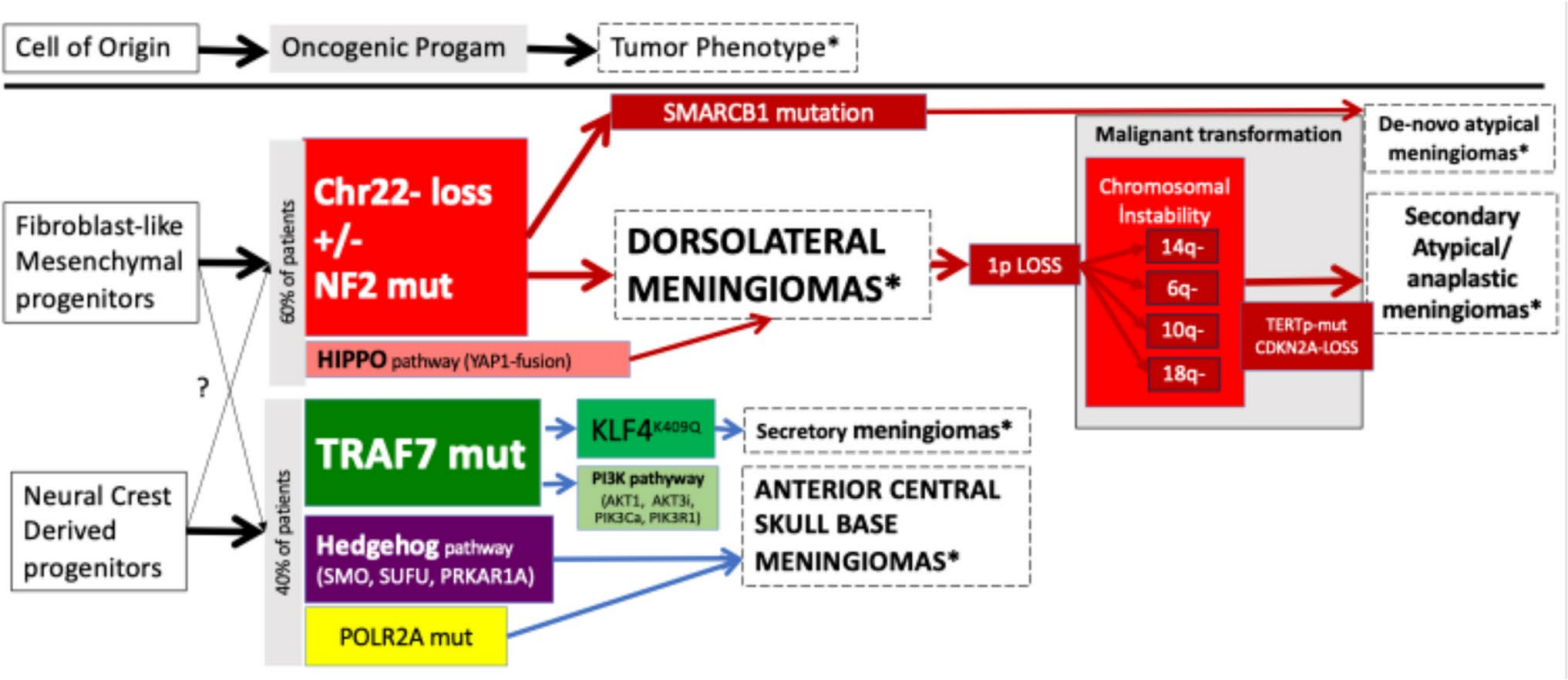
Meningioma is not a single tumour entity but rather an umbrella term used for a group of tumours located at the meninges. Molecular genetic analyses have demonstrated that there are several molecular subsets driven by distinct genetic alterations, which strongly correlate with the anatomical locations and pathological subtypes. The most important of those is the distinction between “dorsolateral” NF2-driven meningiomas and the “anterior central skull base” meningiomas which have “non-NF2 driven” molecular pathophysiology

The oncogenic program, which is the sum of genetic alterations required to transform the cell of origin, also gives clues on the origin of the tumour. Every distinct tumour in the body has a different set of mutations that are commonly seen in that particular tumour type. The last 30 years have exponentially increased our understanding of the oncogenic programs in meningiomas. In 1994 Ruttledge et al. [39]*.* demonstrated that 60% of all sporadic meningiomas had complete inactivation of the *NF2* gene which is located at 22q12. A biallelic inactivation of this tumour suppressor gene is required for meningiomas to develop and this can occur due to (familial inherited NF2 tumour predisposition syndrome) or due to (sporadic alterations in the gene in the meningiomas cell of origin). The two genetic hits most commonly occur due to the deletion of one allele of) the entire chromosome 22 or a large portion of it, accompanied by a mutation in the NF2 gene [38]. The term “loss of heterozygosity” in the context of NF2 refers to this loss of one copy. This tumour suppressor gene pattern led to the discovery of the *Neurofibromin/NF2/Merlin* gene, perturbation of the Hippo pathway and the resultant “loss of contact inhibition” as the underlying mechanism. 2013 marked the publication of several translational studies that demonstrated alternative oncogenic programs (such as the TRAF7, KLF4, PI3K, POLR2A, Sonic Hedgehog mutations and YAP1 fusions) [5, 8, 37, 40]. These oncogenic programs were also strongly associated with anatomical localizations and tumour extension patterns [54]. Today it is well established that NF2 driven meningiomas make up the majority and are mostly localized to the calvarium behind the coronal suture, as well as the tentorial, posterior fossa and the spine. It is known that radiation induced meningiomas are also most commonly NF2-driven. Agnihotri et al*.* [1] have shown that such radiation induced cases share the same cell of origin with other NF2 driven meningiomas based on methylome analysis. Most non-NF2 driven meningiomas (TRAF7, Sonic hedgehog or POLR2A) are localized to the anterior midline skull base and the sphenoid wing [54]. These anatomical predilections of various meningioma molecular subsets are also strongly correlated with the ontogeny of these structures (Fig. 2).

### The neural crest and the mesenchyme

The origin of the human meninges is not homogenous. The neural crest origin of the meninges was first suggested by Oberlin [34]. Our understanding grew further with the chick-quail chimeric system studies of Nicole Le Douarin in 1970’s and Wnt1-CRE lineage tracing studies, which demonstrated that the meninges of telencephalon derived from neural crest, meninges around the brainstem from cephalic mesoderm and meninges around the spinal cord from somitic mesoderm [11, 45]. The origin of vascular endothelium from the mesoderm and pericytes from the neural crest adds to this mosaic. Such dual embryonic origin results in regionally distinct meningeal compartments with unique developmental trajectories, gene expression profiles, and potentially tumorigenic susceptibilities and further complicates the search for a cell of origin. Methylome studies indicate the presence of multiple meningioma subsets, which may correspond to different cells of origin [41]. Meningiomas located in anterior central skull base, which originates from the neural crest, are most commonly non-nf2 driven meningiomas and harbour mutations in TRAF7, Hedgehog pathway or POLR2 [17]. Clark et al*.* [9] compared POLR2A-mutant meningiomas vs. other meningiomas or the dura and demonstrated higher super-enhancer binding and gene expression for WNT6 and ZIC1. During embryogenesis WNT6 is secreted by the non-neural dorsal ectoderm and induces the formation of the neural crest and ZIC1 is expressed by meningeal cells of neural crest origin. This study demonstrated that the common hotspot mutations of non-nf2 driven meningiomas (which are most commonly seen in neural crest derivative- midline skull base) increased downstream signalling of neural crest related genes [9].

### Epithelial vs mesenchymal characteristics

Despite the wide adoption of the “arachnoid cap cell theory”, two competing hypotheses on the origin of meningiomas existed in the twentieth century: one proposing an epithelial origin and the other a mesenchymal derivation [25]. This is not unexpected, as meningiomas can exhibit a wide variety of phenotypes corresponding to the 15 meningioma subtypes. Morphologically, fibroblastic, metaplastic and sarcomatous subtypes are reminiscent of the mesenchymal phenotype and exhibit spindle shaped cells, the production of a collagenous stroma. On the contrary, meningothelial meningiomas exhibit an epithelial phenotype with rounded cytology, intercellular junctions, EMA expression. At the most extreme point of this is the secretory meningioma subtype which exhibits metaplasia with microvilli, cilia, intraluminal secretions and immunopositivity for the carcinoembryonic antigen (CEA). Research has indicated that these epithelial vs mesenchymal characteristics are distributed with a rostro-caudal gradient both in the calvarium and in the skull base [49]. Even today it is not known whether these epithelial or mesenchymal phenotypes are deviations from the same tumour origin, as it is in the case of gliosarcomas. In the 1980’s the famous pathologist John Kepes analysed over 1300 meningiomas, where he used electron microscopy and cell culture studies [25]. He observed collagen production and whorl formation, suggesting fibroblast-like behaviour in meningioma cells. In his AANP honorary lecture in 2004, he questioned the exclusivity of arachnoid origins and proposed an “intermediate cell” model straddling epithelial and mesenchymal traits—a vision that anticipates contemporary transcriptomic findings. His early recognition of transitional phenotypes—cells showing both epithelial-like junctions and collagen-producing capacity—was a critical turning point in meningioma biology. Kepes was among the first to postulate that some meningiomas might originate from multipotent mesenchymal-like cells within the dura [25].

### The dural border cell

In 1975 Nabeshima et al*.* [33] identified the “dural border cell” as a distinct layer within the dura. This cell layer is situated between the dura mater’s dense fibrous layer and the arachnoid barrier cell layer and consisted of flat fibroblasts, lacks collagen and lacks tight junctions. Unless physically forced, no clear distinction exists between these cell groups, which have distinct structural and biochemical characteristics. Recent molecular-genetics studies on embryology of the meninges have identified that arachnoid barrier cell layer develops prenatally as a barrier to separate the fenestrated blood vessels of the dura from the immunopriviledged CSF [15]. Although of mesenchymal origin, these arachnoid barrier cells exhibit epithelial-like (epithelioid) junctions (desmosomes, tight-junctions and gap junctions) as well as pinocytic vesicles (consistent with an active barrier/transport) interface, very similar to the arachnoid cap cells [16, 20]. Demonstration of the prostaglandin D2 synthase by Yamashima et al*.* [52] in 1997 allowed for biochemical differentiation of these two layers. PGDS is a characteristic and abundant protein of the cerebrospinal fluid, the PGDS, clearly marked the arachnoid barrier cells. The finding on the PGDS expression led Kalamarides et al*.* to use this characteristic for an experimental strategy and using a (PGDS-promoter driven biallelic inactivation of NF2 gene) during a strict susceptibility period, reported formation of fibroblastic and meningothelial meningiomas from dural border cells and arachnoid barrier cells respectively [23, 36]. This demonstrated that a “PGDS-positive primordial meningeal cell” which exists before development of the three meningeal layers may produce different meningioma subtypes.

### Meningiomas with no meningeal attachment supporting a “common mesenchymal progenitor origin” hypothesis

Intraventricular, sinonasal, otic or pulmonary meningiomas are some of the entities that are difficult to explain with the arachnoid cap cell origin hypothesis. On the contrary the existence of such entities can be more readily explained by a mesenchymal cell of origin.

**Fig. 3:**
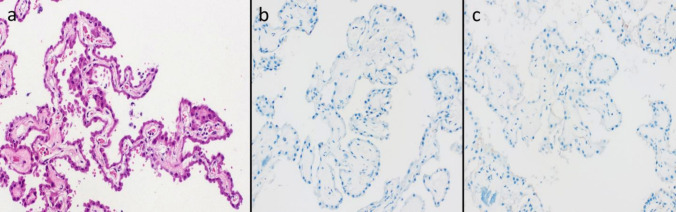
**A** Hematoxylin–eosin (H&E) staining shows the typical architecture of the choroid plexus with papillary fronds lined by a single layer of cuboidal to columnar epithelial cells overlying a delicate fibrovascular core. **B** Immunohistochemical staining for epithelial membrane antigen (EMA) is negative, indicating the absence of arachnoid or meningothelial cell components. **C** Progesterone receptor (PR) immunostaining is negative, further supporting the lack of meningothelial differentiation

Located within the lateral ventricles, often near the trigone, ***intraventricular meningiomas*** emerge in a compartment devoid of meningeal structures. The ventricular system is lined by ependyma but does not contain meningeal tissue. Based on the “arachnoid cap cell origin theory” it was almost universally cited that intraventricular meningiomas arise from meningeal cell remnants that were originally transported during the formation of the choroid plexus. However, there is no observational or experimental data to support this hypothesis. The choroid plexus of the lateral ventricles develops embryologically around 8.5 weeks of gestation by invagination of blood vessels into the medial surface of the hemisphere, pushing through the primitive pia and the ventricular ependyma [47]. The invagination site later remains as the choroid fissure. Later in this process the ependyma is induced to make the choroid plexus epithelium (which is of neuroectodermal origin) and the primitive pia transforms into tela choroidea. Single cell transcriptomics identifies these dorsal thalamic and third ventricular “region specific pial cells” that are CRYM positive but do not express pial(p75NTR) or archnoidal/dural (CRABP2) genes [16]. This suggest that tela choroidea (which is the only remnant of primitive pia in within the ventricles) towards a cell type that derives from a common progenitor but develops into a tissue that is distinct from the meninges. In the paediatric and adult population, the tela choroidea is morphologically fundamentally different from the dura and does not exhibit any structures morphologically or immunohistochemically reminiscent of the arachnoidal cap cell. Recent single cell transcriptomic studies of human choroid plexus have not identified any subset that is reminiscent of arachnoid cap cell [14, 16]. In further support of this finding, we examined 20 surgical, non-neoplastic ChP samples and found no cell groups within ventricular compartments, which resembled the morphology or were immunopositive for arachnoid associated markers, EMA and PR (Unpublished data, Fig. 3).

The ChP develops by an invasion of the arteries into the pia and then ependyma, leaving behind the choroid fissure. The resultant ChP structure has the choroid epithelium (derived from the ependyma, hence neuroectoderm), and the choroid plexus stroma containing vessels and connective tissue (deriving from the cranial mesoderm). Therefore, the embryology of the ChP does not involve the arachnoid. Building on these insight, we demonstrated that intraventricular meningiomas (but not extraventricular meningiomas) expressed OTX2, which is the major transcription factor involved in the choroid plexus development [19]. Junwirth et al*.* [22] demonstrated that 89% of intraventricular meningiomas were NF2 driven, which we also observed in our institutional cohort of IVM, where 8 of 8 tested cases had Chr22 loss. These findings indicate that intraventricular meningiomas, which are like calvarial meningiomas, although there are no meningeal structures and no neural crest derived structures within the ventricles, from which they can arise. A more suitable explanation would be that both intraventricular and extraventricular NF2 driven meningiomas would arise from mesenchymal progenitors.

Another meningioma that has no meningeal attachment is the ***primary pulmonary meningioma***. These rare tumours recapitulate the histological and immunophenotypic features of CNS meningiomas but occur in an organ with no meninges [29]. Their existence reinforces the argument against a simplistic arachnoid cap cell driven hypothesis for meningioma ontology.

### Fibroblast-like progenitors in meningeal compartments: the latest candidate

Growing high-quality evidence suggests that fibroblast-like progenitor cells within the meningeal layers—particularly the dura mater—may serve as the cellular origin for at least for NF2 driven meningiomas. This hypothesis is supported by developmental, ultrastructural, and transcriptomic studies. As mentioned above, meningiomas can exhibit both epithelial and mesenchymal features. Historically, Kepes’ observations in the 1960 s using electron microscopy and tissue culture indicated that the morphology and behaviour of some meningioma cells resembled connective tissue fibroblasts rather than epithelial arachnoid cells. Based on these observations he proposed that mesenchymal/mesodermal progenitor cells within the dura might give rise to meningiomas. However, the technology of the time was not capable of providing the necessary evidence. Advancements in single-cell RNA sequencing (scRNA-seq) and spatial transcriptomics after 2020’s revolutionized our understanding of meningeal cellular diversity, providing unprecedented resolution of lineage-specific gene expression within both normal and neoplastic dura. DeSisto et al*.* [16] performed scRNA-seq of human and murine dura and identified spatially distinct fibroblast subtypes, including perivascular and border-associated fibroblasts with unique gene expression profiles. The study also demonstrated a progressive, ventral to dorsal maturation of the telencephalic meninges. Kearns et al*.* [24] extended these findings to human meningiomas, revealing that tumour cells in certain samples express fibroblast-associated genes (e.g., PDGFRA, LUM, COL1A2) while lacking canonical arachnoid markers such as S100B and AQP1. These fibroblast-like populations were often located near the dura–tumour interface, suggesting a potential site of origin. Vinsland et al*.* [50]. analysed foetal and adult human meninges using spatial transcriptomics and scRNA-seq and found that meningioma cells, transcriptionally resembled dural cells rather than arachnoidal cells. The authors also indicated that common meningioma driver gene expression was localized to the inner dura [50]. Furthermore, spatial transcriptomic overlays confirmed that fibroblast-like tumor cells were often concentrated near the tumor–dura interface, consistent with a dura-based cell of origin. This spatial correlation reinforces the concept that tumor-initiating events may occur within mesodermal derived dural fibroblasts or bipotential precursors, rather than solely within arachnoid villi. This finding is also consistent with the findings of Kalamarides et al. indicating to a precursor of both dural border cells and arachnoid barrier cells as the cell of origin in a NF2 driven transgenic animal model [23]. Choudhury et al*.* [7] reported in their single cell transcriptomic study of 6 meningiomas (with biallelic inactivation of NF2) that such mural cells showed two clusters (one expressing cancer stem cell markers NOTCH3, THY1 and the other expressing markers of cell cycle progression). In this study the authors also traced these NOTCH3 + cells to (PDGFRB or SMA expressing) mural cells in perinatal and adult brain vasculature. However, in IHC studies of adult meninges, such NOTCH3 + cells were adjacent but not overlapping with vascular smooth muscle cells. These findings indicate that, at least in a subset of meningiomas, fibroblast-like, mesenchymal progenitors, which are in the dura may act as the cell of origin for NF2 driven fibrous-meningiomas. It is however, unlikely that the same cell of origin would produce (NF2-driven meningiomas of pure “meningothelial” phenotype) or (non-NF2 driven meningiomas) and this has still to be proven. As can be concluded from aforementioned findings, the cell of origin is not universal, but it varies among different meningiomas.

Additionally, the maturation sequence may also contribute to anatomical variation in meningioma behaviour, despite having the same cell of origin. A ventro-dorsal maturation and spatial transcriptomic heterogeneity has been demonstrated for mesenchymal, fibroblast like progenitors in meninges [26, 49, 54]. Although the fibroblast like mesenchymal progenitor is likely the.

### The role of the meningioma microenvironment

Our understanding of tumour biology in the last century evolved from a tumour-cell centric view to a better understanding of the tumour microenvironment (TME). The most striking example of this was demonstrated for neurofibromas in the context of NF1, where locally recruited, (haploinsufficient) mast cells are hyperstimulated by “Stem Cell Factor” of neoplastic Schwann cell origin, which in turn recruits fibroblasts and endothelial cells to result in the formation of a neurofibroma [53]. The meningioma microenvironment must also be considered when interpreting the findings on the cell of origin. The meningioma microenvironment contains resident/recruited myeloid cells (macrophages), mast cells and infiltrating lymphocytes as well as the parenchymal fibroblasts and vascular cells (endothelial cells and pericytes). This TME has at least two possible effects on the cell of origin: First, the niche can actively “instruct” the cell of origin to result in variations in tumour phenotype [18, 30, 55]. Another aspect of the TME is that it is variable anatomically and can be a confounder during interpretation of experimental findings. The constituents of the TME varies based on anatomical location, tumour biology/molecular biology as well as the treatment (effects of surgery/radiation on the tumour microenvironment) [32, 51]. As the TME is not equal at all anatomical locations, experimental findings can look like surrogate marker for origin, even it is only a surrogate for anatomical location.

## Conclusions

The traditional dogma of arachnoid cap cell origin, though historically important and valuable, is insufficient to explain the histological, molecular, and anatomical heterogeneity of meningiomas. Emerging evidence from developmental biology, spatial transcriptomics, and now proteomics supports that there are likely more than one cell of origin which corresponds to the great variation in meningioma phenotype. The current literature also supports the notion that such cells or origin are likely early progenitors that exist early during development of the meninges, with local environmental cues modulating its phenotype.

## Data Availability

No datasets were generated or analysed during the current study.

## Declarations

The results/data/figures in this manuscript have not been published elsewhere, nor are they under consideration (from one of the contributing authors) by another publisher. The corresponding author has read the journal policies and submit this article in accordance with those policies.

All of the material is owned by the authors and/or no permissions are required.

This manuscript does not report data generation or analysis.

MNP, AED and KÖ have authored the project. AED and KÖ have prepared the figures. All authors have reviewed and edited the final version of the manuscript.

## References

[1] Agnihotri S, Suppiah S, Tonge PD, Jalali S, Danesh A, Bruce JP, Mamatjan Y, Klironomos G, Gonen L, Au K, Mansouri S, Karimi S, Sahm F, von Deimling A, Taylor MD, Laperriere NJ, Pugh TJ, Aldape KD, Zadeh G (2017) Therapeutic radiation for childhood cancer drives structural aberrations of NF2 in meningiomas. Nat Commun 8:186. 10.1038/s41467-017-00174-7 28775249 10.1038/s41467-017-00174-7 PMC5543118

[2] Aoyagi, Kyuno (1912) Ueber die endothelien Zellzapfen in der Dura mater oerebri, und ihre Lokalization in derselben. Neuroglia 11

[3] Bailey P, Bucy PC (1931) The origin and nature of meningeal tumors. Am J Cancer 15:15–54. 10.1158/ajc.1931.15

[4] Barbieri F, Bajetto A, Dellacasagrande I, Solari A, Wurth R, Fernandez V, Rancati S, Ceresa D, Appolloni I, De Luca G, Dono M, Nozza P, Schiapparelli P, Gambaro M, Fiaschi P, Gaggero G, Costanzo N, Thellung S, Malatesta P, Pagano A, Zona G, De Pietri Tonelli D, Florio T (2023) Stem-like signatures in human meningioma cells are under the control of CXCL11/CXCL12 chemokine activity. Neuro Oncol 25:1775–1787. 10.1093/neuonc/noad076 37074930 10.1093/neuonc/noad076 PMC10547527

[5] Brastianos PK, Horowitz PM, Santagata S, Jones RT, McKenna A, Getz G, Ligon KL, Palescandolo E, Van Hummelen P, Ducar MD, Raza A, Sunkavalli A, Macconaill LE, Stemmer-Rachamimov AO, Louis DN, Hahn WC, Dunn IF, Beroukhim R (2013) Genomic sequencing of meningiomas identifies oncogenic SMO and AKT1 mutations. Nat Genet 45:285–289. 10.1038/ng.2526 23334667 10.1038/ng.2526 PMC3739288

[6] Burrows MT (1925) Is cancer a true disease or merely the result of a condition of change in the general organization of the organism? Radiology 4:407–416. 10.1148/4.5.407

[7] Choudhury A, Cady MA, Lucas CG, Najem H, Phillips JJ, Palikuqi B, Zakimi N, Joseph T, Birrueta JO, Chen WC, Oberheim Bush NA, Hervey-Jumper SL, Klein OD, Toedebusch CM, Horbinski CM, Magill ST, Bhaduri A, Perry A, Dickinson PJ, Heimberger AB, Ashworth A, Crouch EE, Raleigh DR (2024) Perivascular NOTCH3+ stem cells drive meningioma tumorigenesis and resistance to radiotherapy. Cancer Discov 14:1823–1837. 10.1158/2159-8290.CD-23-1459 38742767 10.1158/2159-8290.CD-23-1459 PMC11452293

[8] Clark VE, Erson-Omay EZ, Serin A, Yin J, Cotney J, Ozduman K, Avsar T, Li J, Murray PB, Henegariu O, Yilmaz S, Gunel JM, Carrion-Grant G, Yilmaz B, Grady C, Tanrikulu B, Bakircioglu M, Kaymakcalan H, Caglayan AO, Sencar L, Ceyhun E, Atik AF, Bayri Y, Bai H, Kolb LE, Hebert RM, Omay SB, Mishra-Gorur K, Choi M, Overton JD, Holland EC, Mane S, State MW, Bilguvar K, Baehring JM, Gutin PH, Piepmeier JM, Vortmeyer A, Brennan CW, Pamir MN, Kilic T, Lifton RP, Noonan JP, Yasuno K, Gunel M (2013) Genomic analysis of non-NF2 meningiomas reveals mutations in TRAF7, KLF4, AKT1, and SMO. Science 339:1077–1080. 10.1126/science.1233009 23348505 10.1126/science.1233009 PMC4808587

[9] Clark VE, Harmanci AS, Bai H, Youngblood MW, Lee TI, Baranoski JF, Ercan-Sencicek AG, Abraham BJ, Weintraub AS, Hnisz D, Simon M, Krischek B, Erson-Omay EZ, Henegariu O, Carrion-Grant G, Mishra-Gorur K, Duran D, Goldmann JE, Schramm J, Goldbrunner R, Piepmeier JM, Vortmeyer AO, Gunel JM, Bilguvar K, Yasuno K, Young RA, Gunel M (2016) Recurrent somatic mutations in POLR2A define a distinct subset of meningiomas. Nat Genet 48:1253–1259. 10.1038/ng.3651 27548314 10.1038/ng.3651 PMC5114141

[10] Cleland J (1864) Description of two tumours adherent to the deep surface of the dura mater. Glasg Med J 11:148–159 PMC5801940 30432487

[11] Couly GF, Le Douarin NM (1987) Mapping of the early neural primordium in quail-chick chimeras. II. The prosencephalic neural plate and neural folds: implications for the genesis of cephalic human congenital abnormalities. Dev Biol 120:198–214. 10.1016/0012-1606(87)90118-7 3817289 10.1016/0012-1606(87)90118-7

[12] Cruveilhier J (1849) Traité d’anatomie pathologique générale. J.-B. Ballière, Paris

[13] Cushing H (1922) The “meningiomas.” The Cavendish Lecture. BMJ 24:1001–1002

[14] Dani N, Herbst RH, McCabe C, Green GS, Kaiser K, Head JP, Cui J, Shipley FB, Jang A, Dionne D, Nguyen L, Rodman C, Riesenfeld SJ, Prochazka J, Prochazkova M, Sedlacek R, Zhang F, Bryja V, Rozenblatt-Rosen O, Habib N, Regev A, Lehtinen MK (2021) A cellular and spatial map of the choroid plexus across brain ventricles and ages. Cell 184:3056–3074. 10.1016/j.cell.2021.04.003 33932339 10.1016/j.cell.2021.04.003 PMC8214809

[15] Derk J, Como CN, Jones HE, Joyce LR, Kim S, Spencer BL, Bonney S, O’Rourke R, Pawlikowski B, Doran KS, Siegenthaler JA (2023) Formation and function of the meningeal arachnoid barrier around the developing mouse brain. Dev Cell 58:635-644 e634. 10.1016/j.devcel.2023.03.005 36996816 10.1016/j.devcel.2023.03.005 PMC10231667

[16] DeSisto J, O’Rourke R, Jones HE, Pawlikowski B, Malek AD, Bonney S, Guimiot F, Jones KL, Siegenthaler JA (2020) Single-cell transcriptomic analyses of the developing meninges reveal meningeal fibroblast diversity and function. Dev Cell 54:43-59 e44. 10.1016/j.devcel.2020.06.009 32634398 10.1016/j.devcel.2020.06.009 PMC7769050

[17] Fountain DM, Smith MJ, O’Leary C, Pathmanaban ON, Roncaroli F, Bobola N, King AT, Evans DG (2021) The spatial phenotype of genotypically distinct meningiomas demonstrate potential implications of the embryology of the meninges. Oncogene 40:875–884. 10.1038/s41388-020-01568-6 33262459 10.1038/s41388-020-01568-6 PMC8440207

[18] Fu XH, Li JP, Li XY, Tan Y, Zhao M, Zhang SF, Wu XD, Xu JG (2022) M2-Macrophage-derived exosomes promote meningioma progression through TGF-beta signaling pathway. J Immunol Res 2022:8326591. 10.1155/2022/8326591 35637794 10.1155/2022/8326591 PMC9146444

[19] Gungor A, Danyeli AE, Akbas A, Eksi MS, Guduk M, Ozduman K, Pamir MN (2019) Ventricular meningiomas: surgical strategies and a new finding that suggest an origin from the Choroid Plexus epithelium. World Neurosurg 129:e177–e190. 10.1016/j.wneu.2019.05.092 31121376 10.1016/j.wneu.2019.05.092

[20] Hasegawa M, Yamashima T, Kida S, Yamashita J (1997) Membranous ultrastructure of human arachnoid cells. J Neuropathol Exp Neurol 56:1217–1227. 10.1097/00005072-199711000-00006 9370232 10.1097/00005072-199711000-00006

[21] Ince TA, Richardson AL, Bell GW, Saitoh M, Godar S, Karnoub AE, Iglehart JD, Weinberg RA (2007) Transformation of different human breast epithelial cell types leads to distinct tumor phenotypes. Cancer Cell 12:160–170. 10.1016/j.ccr.2007.06.013 17692807 10.1016/j.ccr.2007.06.013

[22] Jungwirth G, Warta R, Beynon C, Sahm F, von Deimling A, Unterberg A, Herold-Mende C, Jungk C (2019) Intraventricular meningiomas frequently harbor NF2 mutations but lack common genetic alterations in TRAF7, AKT1, SMO, KLF4, PIK3CA, and TERT. Acta Neuropathol Commun 7:140. 10.1186/s40478-019-0793-4 31470906 10.1186/s40478-019-0793-4 PMC6716845

[23] Kalamarides M, Stemmer-Rachamimov AO, Niwa-Kawakita M, Chareyre F, Taranchon E, Han ZY, Martinelli C, Lusis EA, Hegedus B, Gutmann DH, Giovannini M (2011) Identification of a progenitor cell of origin capable of generating diverse meningioma histological subtypes. Oncogene 30:2333–2344. 10.1038/onc.2010.609 21242963 10.1038/onc.2010.609

[24] Kearns NA, Iatrou A, Flood DJ, De Tissera S, Mullaney ZM, Xu J, Gaiteri C, Bennett DA, Wang Y (2023) Dissecting the human leptomeninges at single-cell resolution. Nat Commun 14:7036. 10.1038/s41467-023-42825-y 37923721 10.1038/s41467-023-42825-y PMC10624900

[25] Kepes JJ (1986) Presidential address: the histopathology of meningiomas. A reflection of origins and expected behavior? J Neuropathol Exp Neurol 45:95–107 3005518

[26] Kocyigit S, Chavez MM, Orhun O, O’Brien J, Inan A, Yasar AH, Dincer A, Moliterno J, Gunel M, Pamir MN, Ozduman K, Ersen-Danyeli A (2025) Posterior parasagittal meningiomas display aggressive features independent of size: a multicenter analysis. J Neurooncol 175:101–110. 10.1007/s11060-025-05103-z 40569492 10.1007/s11060-025-05103-z PMC12367906

[27] Lebert H (1851) Traité Pratique des Maladies Cancéreuses et des Affections Curables Confondus avec Ie Cancer. J.B. Bailliére, Paris

[28] Lim HY, Kim KM, Kim BK, Shim JK, Lee JH, Huh YM, Kim SH, Kim EH, Park EK, Shim KW, Chang JH, Kim DS, Kim SH, Hong YK, Lee SJ, Kang SG (2013) Isolation of mesenchymal stem-like cells in meningioma specimens. Int J Oncol 43:1260–1268. 10.3892/ijo.2013.2053 23921459 10.3892/ijo.2013.2053

[29] Liu X, Liu J, Nai T, Yang Y, Hu Y (2023) Primary ectopic meningioma in the thoracic cavity: a rare case report and review of the literature. Front Oncol 13:1149627. 10.3389/fonc.2023.1149627 37114141 10.3389/fonc.2023.1149627 PMC10126498

[30] Lotsch C, Warta R, Liu F, Jungwirth G, Rommel C, Barthel M, Lamszus K, Kessler AF, Grabe N, Loehr M, Ketter R, Senft C, Maas SLN, Sievers P, Westphal M, Krieg SM, Unterberg A, Simon M, von Deimling A, Sahm F, Raleigh DR, Herold-Mende C (2025) Tumor-associated macrophages in meningiomas: a novel biomarker for poor survival outperforming the benefits of T cells. Acta Neuropathol 150:41. 10.1007/s00401-025-02948-6 41065822 10.1007/s00401-025-02948-6 PMC12511222

[31] Louis A (1774) Memoire sur les tumeurs fongueuses de la dure-mere. Mem Acad R Chir 13:9–46

[32] Lucas CG, Mirchia K, Seo K, Najem H, Chen WC, Zakimi N, Foster K, Eaton CD, Cady MA, Choudhury A, Liu SJ, Phillips JJ, Magill ST, Horbinski CM, Solomon DA, Perry A, Vasudevan HN, Heimberger AB, Raleigh DR (2024) Spatial genomic, biochemical and cellular mechanisms underlying meningioma heterogeneity and evolution. Nat Genet 56:1121–1133. 10.1038/s41588-024-01747-1 38760638 10.1038/s41588-024-01747-1 PMC11239374

[33] Nabeshima S, Reese TS, Landis DM, Brightman MW (1975) Junctions in the meninges and marginal glia. J Comp Neurol 164:127–169. 10.1002/cne.901640202 810497 10.1002/cne.901640202

[34] Oberlin (1931) Rechrchez sur l’origine des meninges craniennes ches les vertebres. Journal D l’Anatomie-Paris

[35] Patil C, Laws E (2010) Meningioma: History of the tumor and its management. In: Pamir MN, Black PM, Fahlbusch R (eds) Meningiomas: A comprehensive text. Saunders/Elsevier, Philadelphia, pp 3–11

[36] Peyre M, Salaud C, Clermont-Taranchon E, Niwa-Kawakita M, Goutagny S, Mawrin C, Giovannini M, Kalamarides M (2015) PDGF activation in PGDS-positive arachnoid cells induces meningioma formation in mice promoting tumor progression in combination with Nf2 and Cdkn2ab loss. Oncotarget 6:32713–32722. 10.18632/oncotarget.5296 26418719 10.18632/oncotarget.5296 PMC4741724

[37] Reuss DE, Piro RM, Jones DT, Simon M, Ketter R, Kool M, Becker A, Sahm F, Pusch S, Meyer J, Hagenlocher C, Schweizer L, Capper D, Kickingereder P, Mucha J, Koelsche C, Jager N, Santarius T, Tarpey PS, Stephens PJ, Futreal P A, Wellenreuther R, Kraus J, Lenartz D, Herold-Mende C, Hartmann C, Mawrin C, Giese N, Eils R, Collins VP, Konig R, Wiestler OD, Pfister SM, von Deimling A (2013) Secretory meningiomas are defined by combined KLF4 K409Q and TRAF7 mutations. Acta Neuropathol 125:351–358. 10.1007/s00401-013-1093-x 23404370 10.1007/s00401-013-1093-x

[38] Ruttledge MH, Rouleau GA (2005) Role of the Neurofibromatosis Type 2 gene in the development of tumors of the nervous system. Neurosurg Focus 19:E6. 10.3171/foc.2005.19.5.7 16398470 10.3171/foc.2005.19.5.7

[39] Ruttledge MH, Sarrazin J, Rangaratnam S, Phelan CM, Twist E, Merel P, Delattre O, Thomas G, Nordenskjold M, Collins VP et al (1994) Evidence for the complete inactivation of the NF2 gene in the majority of sporadic meningiomas. Nat Genet 6:180–184. 10.1038/ng0294-180 8162072 10.1038/ng0294-180

[40] Sahm F, Bissel J, Koelsche C, Schweizer L, Capper D, Reuss D, Bohmer K, Lass U, Gock T, Kalis K, Meyer J, Habel A, Brehmer S, Mittelbronn M, Jones DT, Schittenhelm J, Urbschat S, Ketter R, Heim S, Mawrin C, Hainfellner JA, Berghoff AS, Preusser M, Becker A, Herold-Mende C, Unterberg A, Hartmann C, Kickingereder P, Collins VP, Pfister SM, von Deimling A (2013) AKT1E17K mutations cluster with meningothelial and transitional meningiomas and can be detected by SFRP1 immunohistochemistry. Acta Neuropathol 126:757–762. 10.1007/s00401-013-1187-5 24096618 10.1007/s00401-013-1187-5

[41] Sahm F, Schrimpf D, Stichel D, Jones DTW, Hielscher T, Schefzyk S, Okonechnikov K, Koelsche C, Reuss DE, Capper D, Sturm D, Wirsching HG, Berghoff AS, Baumgarten P, Kratz A, Huang K, Wefers AK, Hovestadt V, Sill M, Ellis HP, Kurian KM, Okuducu AF, Jungk C, Drueschler K, Schick M, Bewerunge-Hudler M, Mawrin C, Seiz-Rosenhagen M, Ketter R, Simon M, Westphal M, Lamszus K, Becker A, Koch A, Schittenhelm J, Rushing EJ, Collins VP, Brehmer S, Chavez L, Platten M, Hanggi D, Unterberg A, Paulus W, Wick W, Pfister SM, Mittelbronn M, Preusser M, Herold-Mende C, Weller M, von Deimling A (2017) DNA methylation-based classification and grading system for meningioma: a multicentre, retrospective analysis. Lancet Oncol 18:682–694. 10.1016/S1470-2045(17)30155-9 28314689 10.1016/S1470-2045(17)30155-9

[42] Scherer HJ (1940) A critical review: the pathology of cerebral gliomas. J Neurol Neurosurg Psychiatry 3:147–177. 10.1136/jnnp.3.2.147 10.1136/jnnp.3.2.147 PMC1088179 21610973

[43] Schmidt MB (1902) Ueber die Pacchioni’schen Granulationen und ihr Verhältniss zu den Sarcomen und Psammomen der Dura mater. Archiv für pathologische Anatomie und Physiologie und für klinische Medicin( Virchow’s Achiv) 170:429–464. 10.1007/BF01948415

[44] Shih C, Padhy LC, Murray M, Weinberg RA (1981) Transforming genes of carcinomas and neuroblastomas introduced into mouse fibroblasts. Nature 290:261–264. 10.1038/290261a0 7207618 10.1038/290261a0

[45] Siegenthaler JA, Ashique AM, Zarbalis K, Patterson KP, Hecht JH, Kane MA, Folias AE, Choe Y, May SR, Kume T, Napoli JL, Peterson AS, Pleasure SJ (2009) Retinoic acid from the meninges regulates cortical neuron generation. Cell 139:597–609. 10.1016/j.cell.2009.10.004 19879845 10.1016/j.cell.2009.10.004 PMC2772834

[46] Singh SK, Hawkins C, Clarke ID, Squire JA, Bayani J, Hide T, Henkelman RM, Cusimano MD, Dirks PB (2004) Identification of human brain tumour initiating cells. Nature 432:396–401. 10.1038/nature03128 15549107 10.1038/nature03128

[47] Strong LH (1964) The vascular and ependymal development of the early stages of the tela choroidea of the lateral ventricle of the mammal. J Morphol 114:59–82. 10.1002/jmor.1051140104 14114963 10.1002/jmor.1051140104

[48] Thirimanne HN, Almiron-Bonnin D, Nuechterlein N, Arora S, Jensen M, Parada CA, Qiu C, Szulzewsky F, English CW, Chen WC, Sievers P, Nassiri F, Wang JZ, Klisch TJ, Aldape KD, Patel AJ, Cimino PJ, Zadeh G, Sahm F, Raleigh DR, Shendure J, Ferreira M, Holland EC (2024) Meningioma transcriptomic landscape demonstrates novel subtypes with regional associated biology and patient outcome. Cell Genom 4:100566. 10.1016/j.xgen.2024.100566 38788713 10.1016/j.xgen.2024.100566 PMC11228955

[49] Ulgen E, Bektasoglu PK, Sav MA, Can O, Danyeli AE, Hizal DB, Pamir MN, Ozduman K (2019) Meningiomas display a specific immunoexpression pattern in a rostrocaudal gradient: an analysis of 366 patients. World Neurosurg 123:e520–e535. 10.1016/j.wneu.2018.11.201 30503291 10.1016/j.wneu.2018.11.201

[50] Vinsland E, Salas SM, Kapustova I, Hu L, Webb S, Li X, He X, Nilsson M, Haniffa M, Barker R, Persson O, Raleigh DR, Sundstrom E, Lonnerberg P, Linnarsson S (2025) Cell atlas of the developing human meninges reveals a dura origin of meningioma. bioRxiv. 10.1101/2025.07.08.663122 40672210 10.1101/2025.07.08.663122 PMC12265625

[51] White AJ, Harary M, Casaos J, Everson RG (2024) Current immunotherapy techniques in meningioma. Expert Rev Anticancer Ther 24:931–941. 10.1080/14737140.2024.2399252 39233324 10.1080/14737140.2024.2399252

[52] Yamashima T, Sakuda K, Tohma Y, Yamashita J, Oda H, Irikura D, Eguchi N, Beuckmann CT, Kanaoka Y, Urade Y, Hayaishi O (1997) Prostaglandin D synthase (beta-trace) in human arachnoid and meningioma cells: roles as a cell marker or in cerebrospinal fluid absorption, tumorigenesis, and calcification process. J Neurosci 17:2376–2382. 10.1523/JNEUROSCI.17-07-02376.1997 9065498 10.1523/JNEUROSCI.17-07-02376.1997 PMC6573504

[53] Yang FC, Ingram DA, Chen S, Zhu Y, Yuan J, Li X, Yang X, Knowles S, Horn W, Li Y, Zhang S, Yang Y, Vakili ST, Yu M, Burns D, Robertson K, Hutchins G, Parada LF, Clapp DW (2008) Nf1-dependent tumors require a microenvironment containing Nf1+/- - and c-kit-dependent bone marrow. Cell 135:437–448. 10.1016/j.cell.2008.08.041 18984156 10.1016/j.cell.2008.08.041 PMC2788814

[54] Youngblood MW, Duran D, Montejo JD, Li C, Omay SB, Ozduman K, Sheth AH, Zhao AY, Tyrtova E, Miyagishima DF, Fomchenko EI, Hong CS, Clark VE, Riche M, Peyre M, Boetto J, Sohrabi S, Koljaka S, Baranoski JF, Knight J, Zhu H, Pamir MN, Avsar T, Kilic T, Schramm J, Timmer M, Goldbrunner R, Gong Y, Bayri Y, Amankulor N, Hamilton RL, Bilguvar K, Tikhonova I, Tomak PR, Huttner A, Simon M, Krischek B, Kalamarides M, Erson-Omay EZ, Moliterno J, Gunel M (2020) Correlations between genomic subgroup and clinical features in a cohort of more than 3000 meningiomas. J Neurosurg 133:1345–1354. 10.3171/2019.8.JNS191266 31653806 10.3171/2019.8.JNS191266

[55] Zhang T, Adams CL, Fejer G, Ercolano E, Cutajar J, Na J, Sahm F, Hanemann CO (2025) Tumour-associated macrophage infiltration differs in meningioma genotypes, and is important in tumour dynamics. J Exp Clin Cancer Res 44:162. 10.1186/s13046-025-03419-2 40420192 10.1186/s13046-025-03419-2 PMC12107748

